# Evaluation of the Th17 pathway in psoriasis and geographic tongue^☆^^☆☆^

**DOI:** 10.1016/j.abd.2019.01.006

**Published:** 2019-10-24

**Authors:** Bruna Lavinas Sayed Picciani, Tábata Alves Domingos, Thays Teixeira-Souza, Anna Karoline Fausto-Silva, Eliane Pedra Dias, Sueli Carneiro

**Affiliations:** a Department of Pathology, Faculdade de Medicina, Universidade Federal Fluminense, Niterói, RJ, Brazil; b Graduate Program in Pathology, Faculdade de Medicina, Universidade Federal Fluminense, Niterói, RJ, Brazil; c Sector of Dermatology, Medical Clinic Department, Universidade Federal do Rio de Janeiro, Rio de Janeiro, RJ, Brazil

**Keywords:** Glossitis, Benign migratory, Psoriasis, Th17 cells

## Abstract

**Background:**

Psoriasis is a skin-articular disease with unclear etiopathogenesis. It has been suggested that the disease is immune-mediated by T-lymphocytes, predominantly Th17 cells. Similar to psoriasis, geographic tongue is an inflammatory disease with participation of Th17 cells and direct correlation with psoriasis.

**Objective:**

To investigate and compare the inflammatory responses and the Th17 pathway in psoriasis and geographic tongue.

**Methods:**

This was a cross-sectional study with 46 participants that were categorized into three groups: (A) patients with psoriasis vulgaris; (B) patients with geographic tongue and psoriasis; (C) patients with geographic tongue without psoriasis. All patients underwent physical examination, and a skin and oral biopsy for histopathological examination and immunohistochemical analysis with anti-IL6, anti-IL17, and anti-IL23 antibodies.

**Results:**

Histological analysis of all lesions showed mononuclear inflammatory infiltrate. However, moderate intensity was prevalent for the patients with geographic tongue and psoriasis and geographic tongue groups. Immunopositivity for the antibodies anti-IL6, anti-IL17, and anti-IL23 revealed cytoplasmic staining, mainly basal and parabasal, in both psoriasis and geographic tongue. Regarding IL-6, in patients with geographic tongue and psoriasis cases the staining was stronger than in patients with geographic tongue without psoriasis cases. IL-17 evidenced more pronounced and extensive staining when compared to the other analyzed interleukins. IL-23 presented similar immunopositivity for both geographic tongue and psoriasis, demonstrating that the neutrophils recruited into the epithelium were stained.

**Study limitation:**

This study was limited by the number of cases.

**Conclusion:**

The inflammatory process and immunostaining of IL-6, IL-17, and IL-23 were similar in geographic tongue and psoriasis, suggesting the existence of a type of geographic tongue that represents an oral manifestation of psoriasis.

## Introduction

Psoriasis is a chronic immune-mediated inflammatory disease that affects 1% to 3% of the population worldwide.^1^ Although its etiology is unknown, it is believed that it is an immunological disease with genetic basis, being characterized by an inappropriate immunological activation triggered by T-lymphocytes, predominantly Th1 and Th17.^2^, ^3^ Interleukins (IL) 17 and 23 are responsible for the development and maintenance of Th17 cells, acting in production of IL-22 and IL-6, which stimulate keratinocyte proliferation. These findings regarding high levels of interleukins in the skin lesions of psoriasis patients provide tools for increasing interest about IL-23 and Th17 in psoriasis.^2^, ^4^, ^5^

Psoriasis presents variable expressivity according to the genetic component and environmental factors, which could affect other locations such as the oral mucosa.^6^, ^7^, ^8^ The oral lesion most strongly associated with psoriasis is geographic tongue (GT), characterized by irregular areas of loss of filiform papillae, often circumscribed by a yellowish-white line.^8^, ^9^, ^10^ Similar to psoriasis, GT is a chronic inflammatory disease with a genetic and immunological basis that has been described to have also a role of IL-6 and IL-17 in its development.^10^, ^11^, ^12^, ^13^

The occurrence of oral manifestations of psoriasis is seldom reported in the literature; its clinical, histological, and immunogenetic similarities with GT creates controversial thoughts about the actual existence of an oral lesion caused by psoriasis or whether GT might be a marker which indicates the severity of skin disease, since these lesions are found more often in severe psoriasis.^8^, ^9^, ^14^, ^15^, ^16^, ^17^, ^18^, ^19^ Thus, further studies are needed to better understand the pathogenesis and association between theses conditions.

The aim of this study was to investigate and compare inflammatory responses and the Th17 pathway through evolution of the expression of IL-6, IL-17, and IL-23 in psoriasis and GT.

## Methods

This was a cross-sectional study, with 46 participants aged greater than 18 years, with clinical diagnosis of psoriasis vulgaris and GT. Based on the dermatological and stomatological examinations, the participants were categorized into three groups: (A) PV, consisting of 11 patients with psoriasis vulgaris; (B) PV-GT, consisting of 15 patients with GT and psoriasis vulgaris; and (C) NPS, consisting of 20 patients with GT without psoriasis. The subjects were excluded if they had other skin diseases, autoimmune diseases, metabolic diseases, severe cardiovascular diseases, infections by hepatitis virus B or C or HIV, use of immunosuppressive medications, allergies, or possibility of a pregnancy at the time of evaluation.

All patients underwent anamnesis; physical examination and a 5-mm skin punch and oral biopsy for histopathological examination and immunohistochemical analysis of anti-IL-6, anti-IL-17, and anti-IL-23 antibodies. The selection of the biopsy area was based on physical examination. Patients with psoriasis vulgaris had the biopsy performed on typical lesions, which are well-demarcated erythematous-desquamative plaques with silvery scales. Patients with GT had the biopsy performed in areas with white border and the atrophic area. After surgical removal, the tissue fragments were fixed in 10% phosphate-buffered formaldehyde and included in paraffin blocks. A glass slide stained with hematoxylin and eosin was prepared for histopathological analysis of the inflammatory response.

The slides stained with hematoxylin and eosin were scanned at 40× magnification using an ultra-resolution ScanScope™ system (Aperio Technologies – CA, United States) and analyzed with detailed description of all histological alterations. A qualitative and quantitative analysis was conducted, and the predominant cell type (polymorphonuclear or mononuclear) and intensity of inflammatory infiltrate were evaluated. For intensity classification, all scanned slides were analyzed and the one with the most intense inflammatory infiltrate was selected. At 40× magnification, the most cellular area was captured and the cells were manually counted using an auxiliary grid, totalling 360 cells. Thereby, this total number was divided by three, establishing that at 40× magnification the inflammatory infiltrate of the most cellular area would be considered mild if under 120 cells, moderate if between 120 and 240 cells, and intense if above 240 cells.

Subsequently, tissue fragments from paraffin blocks were organized into tissue macroarray (TMaA) paraffin blocks to perform immunohistochemistry (IHC) and further analyses.

The study was approved by the Research Ethics Committee (02330258000-10), and all participants signed an informed consent.

### TMaA construction

To construct the TMaA blocks, donor paraffin blocks containing skin fragments were grouped according to the psoriasis type. Fragments were removed from the donor blocks, placed in identified cassettes, and immersed in a liquid paraffin bath at 57 °C. Metallic molds were prepared to receive the identified fragments. For this, a thin layer of liquid paraffin was placed on a metallic mold for the tissue's adhesion. After the attachment of the tissues on the metallic mold, using anatomical forceps, liquid paraffin was gently inserted to construct the paraffin blocks. Three TMaA paraffin blocks were constructed; the donor paraffin blocks. For each paraffin block, 5-μm sections were collected on positively charged glass slides for IHC.^20^

### IHC

Expression of IL-6, IL-17, and IL-23 was demonstrated immunohistochemically using the immunoperoxidase technique. Primary antibodies used were: mouse monoclonal anti-human IL-6 (Abcam, ab9324; United States; dilution 1:8000), mouse monoclonal anti-human IL-17 (Abcam, ab79056; United States; dilution 1:1500), and mouse monoclonal anti-human IL-23 (Abcam, ab115759; United States; dilution 1:1000). After deparaffinization and rehydration, endogenous peroxidase activity was blocked with 3% hydrogen peroxide for 30 min. Antigen retrieval was performed with citrate buffer (pH 6.0) in a water bath (96 °C) for 40 min. Non-specific protein binding was blocked with serum-free protein block (for 15 min). Sections were incubated for 10 h in a moist chamber at 4 °C with the primary antibodies. The slides were subsequently incubated with the Dako EnVision+Dual Link System-HRP (Dako, K4061; United States) for 30 min. Visualization was performed by incubation for 3 min in a liquid 3,3′diaminobenzidine chromogen system (Dako, K3468;). Sections were counterstained with Harris hematoxylin. A tonsil fragment was used as positive control for the antibody anti-IL-6, lymph node for anti-IL-17, and kidney for anti-IL-23. Negative controls were performed by omission of the primary antibody.

### IHC analysis

Digital images of IHC-stained TMaA slides were obtained at 40× magnification using the Aperio CS2 scanner (Leica Biosystems – United States). After that, the sections were visualized by ImageScope™ software (Leica Biosystems – United States) with subsequent analysis of all fragments. The protein expression of IL-6, IL-17, and IL-23 was shown by the cytoplasmic-positive marking on keratinocytes, inflammatory cells, endothelial cells, and fibroblasts. The immunohistochemistry analysis was conducted in a qualitative and quantitative way, considering the pattern, localization, intensity, and extension of staining. Areas unsuitable for analysis, such as tissue-folds and other artifacts, were excluded.

### Statistical analysis

All statistical tests were performed using SPSS v. 22.0. Categorical variables were reported as proportions, and numeric variables were shown as median, mean, standard deviation (SD), and range. To evaluate the difference among two or more categorical variables, Fisher's exact test was applied. The level of statistical significance for all analyses was set at 5% (*p* < 0.05).

## Results

The age of the 46 participants included in this study ranged between 18 and 85 years, with an average of 52 years (SD = 15), showing a slight preference for the female sex, with 27 (58%) cases.

**Figure 1:**
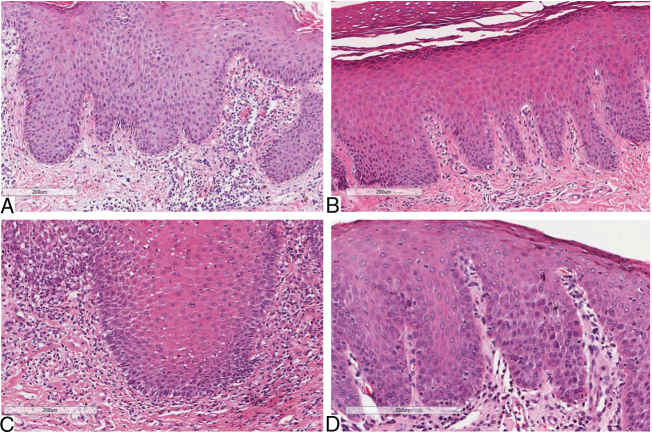
Histopathological aspects of inflammatory infiltrate (Hematoxylin & eosin, x40): A, Psoriasis with moderate infiltrate; B, Psoriasis with mild infiltrate; C, Geographic tongue in psoriatic patient with moderate infiltrate; D, Geographic tongue with mild infiltrate.

Considering skin lesions of psoriatic patients, the superficial inflammatory infiltrate was predominantly mononuclear in 100% of cases. The infiltrate intensity varied among the patients, being moderate in six (54%) patients, mild in three (37%), and intense in two (19%). The comparative analysis of histological criteria between GT and skin lesions in patients with and without psoriasis revealed that both lesions presented inflammatory infiltrate, predominantly mononuclear. However, moderate intensity predominated in patients with GT and psoriasis, and patients exclusively with psoriasis; *p* = 0.078 (Table 1) (Fig. 1).

**Table 1:** Histological aspects of oral and cutaneous lesions of patients presenting geographic tongue, geographic tongue and psoriasis, or isolated psoriasis.

Histological aspects	Geographic tongue without psoriasis, *n* = 20 (%)	Geographic tongue with psoriasis, *n* = 15 (%)	Psoriasis, *n* = 11 (%)	*p*^a^
Superficial inflammatory infiltrate	–	–	–	0.514
Mononuclear	19 (95%)	15 (100%)	11 (100%)	
Polymorphonuclear	1 (5%)	–	–	
Inflammatory infiltrate intensity	–	–	–	0.078
Mild	9 (45%)	2 (13%)	3 (27%)	
Moderate	5 (25%)	11 (74%)	6 (55%)	
Intense	6 (30%)	2 (13%)	2 (18%)	

a Fisher's exact test.

The immunostaining of the three antibodies (anti-IL-6, anti-IL-17, and anti-IL-23) presented a similar cytoplasmic staining pattern, predominantly basal and parabasal, in both GT and psoriasis. When the basal layer was significantly more hyperplasic, the staining was more prominent and extensive. The mononuclear inflammatory infiltrate showed the same pattern of staining for all used antibodies.

**Figure 2:**
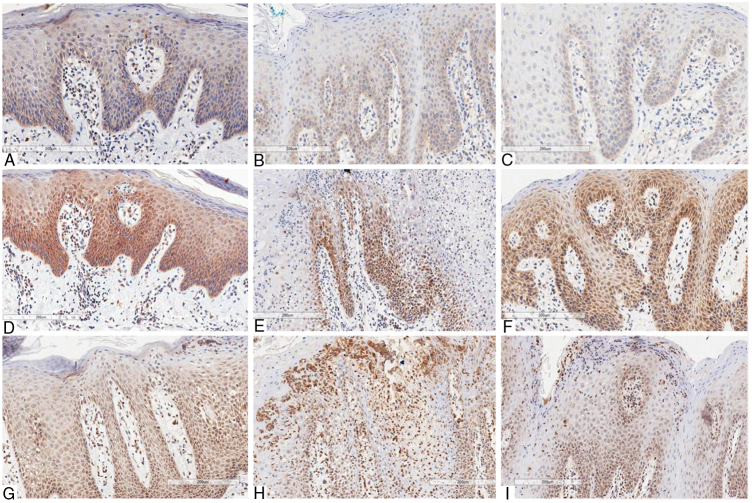
Immunohistochemical marking aspects of interleukins in psoriasis and geographic tongue: Interleukin-6 – A, Psoriasis; B, Geographic tongue in psoriatic patient; C, Geographic tongue. Interleukin-17 – D, Psoriasis; E, Geographic tongue in psoriatic patient; F, Geographic tongue. Interleukin-23 – G, Psoriasis; H, Geographic tongue in psoriatic patient; I, Geographic tongue.

Concerning IL-6, in skin lesions there was immunopositivity in the basal third of the epithelium, being proportional to hyperplasia of the basal layer. The epithelial staining was stronger in regions presenting more intense hyperkeratosis. The immunopositivity in the dermis was seen in mononuclear inflammatory infiltrate, especially lymphocytes, as well as in the vascular endothelium. The staining was more intense in GT patients with psoriasis than in those with GT and without psoriasis. This might be because the former presented more prominent basal-layer hyperplasia, resembling the skin pattern (Fig. 2).

IL-17 presented more evident and extensive staining when compared to the other interleukins evaluated. In skin lesions there was immunopositivity in the two inferior thirds of epidermis, even though some areas showed extension to the superficial layer due to intense hyperplasia. The staining pattern was more homogeneous, intense, and extensive in all fragments when compared to anti-IL-6 staining. In GT patients with psoriasis, the immunostaining pattern was similar to that in GT patients non-affected by psoriasis. However, this staining was less intense than in the skin lesions, and there was no significant difference between the two patterns, as was found for IL-6 staining. In non-psoriatic patients with GT, the immunostaining pattern found was more similar to that found in skin lesions (Fig. 2).

IL-23 presented basal and suprabasal immunopositivity in skin lesions, extending to the superficial layers from the epithelium and being more intense and extensive than IL-6 staining. The neutrophils infiltrated into the epithelium (Munro's microabscesses) were also stained with this antibody. In the dermis, there was positivity in the mononuclear infiltrate and endothelium, similar to the other antibodies used. In GT patients, either with or without psoriasis, the staining pattern was substantially the same as that found in psoriasis lesions, with basal and suprabasal predominance, more intense and extensive than that of IL-6; the neutrophils in the epithelium were also stained, though less intensely than in skin staining (Fig. 2).

## Discussion

Psoriasis is a common skin-articular disease, immunologically triggered by T-lymphocytes, with production of cytokines types Th1 and Th17.^1^, ^3^ The Th17 pathway has been associated with immunopathogenesis of psoriasis and other immune-mediated diseases such as GT.^13^ Several authors suggest an association between these two conditions. Besides the high prevalence in psoriatic patients, GT has clinical, etiologic, microscopic, and genetic similarities with psoriasis.^8^, ^9^, ^14^, ^15^, ^16^, ^17^, ^18^, ^19^ As some patients present GT with no psoriasis, it is difficult for some authors to accept this oral condition as a manifestation of psoriasis.^18^, ^21^, ^22^, ^23^ Moreover, there are only a few studies on the histological and immunohistochemical aspects of GT and its correlation with the skin disease. In the reviewed literature, there were no studies evaluating the inflammatory aspects of GT compared to psoriasis.

The microscopic findings of psoriasis are very characteristic, such as the following: regular increase of the spinous layer with thickening of the lower portions; thickening and swelling of the papillae; parakeratosis; absence of the granular layer; presence of Munro's microabscesses; suprapapillary hypotrophy, occasionally presenting a small spongiform pustule; and inflammatory cell infiltration, mainly T-lymphocytes, macrophages, and neutrophils, in the dermis and submucosa.^8^, ^24^, ^25^ Such features can be also found in GT.^8^, ^26^ The present study proposes a comparative analysis focused on inflammatory response in which the type and intensity of inflammatory infiltrate was evaluated in GT and psoriasis. Both conditions presented mononuclear inflammatory infiltrate predominance, and moderate intensity was prevalent in psoriasis and in psoriatic patients with GT. The inflammatory infiltrate has been highlighted in psoriasis, being particularly composed by T-lymphocytes.^27^ Also, the present authors have not found studies presenting this analysis in the literature encountered.

In 2001, Femiano performed a histological evaluation, comparing GT lesions in psoriatic and non-psoriatic patients. The former group presented analyses histologically compatible with psoriasis. In the latter group, the same characteristics were observed in 80% of the individuals. Thus, the author considered GT as an oral manifestation of psoriasis, but suggested the possibility of having another type of GT non-associated with psoriasis.^26^ Although the histologic differences found in the present study are discreet, it corroborates the theory formulated by Femiano, in 2001, of another type of GT in non-psoriatic patients.

The immunohistochemical analysis was directed to the immunoreactivity of antibodies anti-IL-6, anti-IL-17, and anti-IL-23 in both GT and psoriasis. In the present study, this analysis evidenced similarity in the distribution and quantity of immunopositive cells in both skin and oral lesions, reinforcing the association between them.

In 2000, Goodman et al. evaluated the expression of IL-6 in psoriatic lesional, non-lesional, and normal tissues, revealing higher expression in lesional psoriatic skin than in non-lesional areas and normal skin. Additionally, predominance of IL-6 was observed in the papillae, superficial vascular plexus, and epidermal keratinocytes.^28^ In 1989, Grossman et al. evidenced immunoreactivity for anti-IL-6 in keratinocytes, endothelial cells, and in most of the dermal infiltrate into psoriasis plaques. There was cytoplasmic staining in all cases, and in some cases there was also nuclear and membrane staining. The keratinocytes from the basal layer were more intensely stained than those from the spinous layer.^29^ The present study revealed similarities among the three groups that presented the same pattern of immunopositivity in endothelial cells, keratinocytes, and dermal inflammatory infiltrate, showing cytoplasmic staining, predominantly in the basal and suprabasal layers. In 2014, Alikhani et al. analyzed TNF-α and IL-6 concentration in the saliva of patients with GT and showed a significant increase in these cytokines in this lesion. Those authors concluded that GT is an inflammatory and immune-mediated condition, reinforcing its association with psoriasis.^11^

About 15 years ago, the expression of IL-17 was detected in skin fragments of psoriatic patients, whilst it was not evidenced in normal control tissues, suggesting the involvement of IL-17 in psoriasis immunopathogenesis.^30^ IL-17 is the homonymous cytokine produced by the Th17 subgroups of T-lymphocytes.^4^, ^30^

In 2010, Li Zhang et al. conducted an immunohistochemical analysis of IL-17 in 24 skin fragments from psoriasis, confirming this interleukin was significantly higher in these patients than in healthy individuals, being correlated to the disease severity. That study indicates IL-17 as a pro-inflammatory cytokine able to amplify the development of cutaneous inflammation and maintain the support of chronic dermatitis through keratinocyte stimulation.^31^ The current study demonstrated that IL-17 is present in large numbers in the mononuclear inflammatory infiltrate, besides the immunopositivity in basal and suprabasal keratinocytes from the epithelial hyperplasia and vascular endothelium areas. This occurs because keratinocytes are part of the immune system as well as the endothelial cells, playing a central role in the activation of pathways involved in psoriasis immunopathogenesis, acting firstly as an antigen presenting to T-lymphocytes.^32^, ^33^, ^34^ Herein, the same as in psoriasis, the intense staining of IL-17 in GT demonstrates that this interleukin is also involved in the pathogenesis of this oral lesion. In 2017, Baron reported a case of GT associated with acrodermatitis continua of Hallopeau with good outcome to secukinumab, a selective anti-IL-17A monoclonal antibody.^13^ This finding corroborates with the theory that IL-17 is involved in both conditions.

Regarding IL-23, it is a key cytokine involved in the protective immune response to bacterial and fungal infections, even though its uncontrolled production may also promote inflammatory autoimmunity.^2^, ^35^ Laboratory studies have identified the IL-23/IL-17 axis as the primary signaling pathway leading to characteristic molecular, cellular, and structural changes in psoriatic skin. In the presence of IL-23, secreted mainly from inflammatory dermal dendritic cells (DCs), Th17 cells increase in number and produce large amounts of IL-17, which drive the upregulation of many psoriasis-related genes produced by epidermal keratinocytes. In addition to dermal DCs, epidermal keratinocytes and dermal macrophages are also found to produce IL-23 and may contribute to the pathogenesis and maintenance of psoriatic lesions. New research findings indicate that IL-23 is one of the most important cytokines in the pathogenesis of psoriasis.^2^, ^35^ In 2016, Song et al. demonstrated, by immunohistochemistry, that IL-23 and IL-17 expressions were observed in the epidermis and perivascular dermal area in the psoriasis, suggesting that these interleukins play an important role in this disease.^36^ In the present study, a more intense staining of IL-17 was observed; however, IL-23 also showed similar staining, confirming the link between these two interleukins. In response to IL-23, these Th17 cells create an immune environment that triggers hyperproliferation of keratinocytes and leads to the recruitment of other immune cells.^1^, ^35^ Furthermore, the present study demonstrated – for the first time – the involvement of this interleukin in GT, and the staining of neutrophils with IL-23, mainly in Munro's microabscess areas. It has been described that neutrophils in the initial phase of the psoriatic lesion reach the epidermis, constituting Munro's microabscess. Neutrophils are known for their relevant production of pro-inflammatory mediators and, since these cells express IL-17 receptors, IL-17 may represent an import signal of autocrine auto-amplification. Moreover, neutrophils respond to IL-23, which leads to an increased number of IL-17+ and IL-23+ neutrophils in psoriasis.^2^

## Conclusion

This study shows that the inflammatory response and immunostaining of IL-6, IL-17, and IL-23 are often similar in both GT and psoriasis, reinforcing the possibility that there is a type of GT that represents an oral manifestation of psoriasis. Therefore, the Th17 pathway seems to play a central role in GT, regardless of the presence of psoriasis. Further studies are necessary for a better understanding of the role of the Th17 pathway in the pathogenesis of GT.

## Financial support

FUNADERM – Sociedade Brasileira de Dermatologia/Brazilian Society of Dermatology.

## Author's contribution

Bruna Lavinas Sayed Picciani: Statistical analysis; approval of the final version of the manuscript; conception and planning of the study; elaboration and writing of the manuscript; obtaining, analyzing and interpreting the data; effective participation in research orientation; intellectual participation in propaedeutic and/or therapeutic conduct of the cases studied; critical review of the literature; critical review of the manuscript.

Tábata Alves Domingos: Elaboration and writing of the manuscript; obtaining, analyzing and interpreting the data; critical review of the manuscript.

Thays Teixeira-Souza: Elaboration and writing of the manuscript; obtaining, analyzing and interpreting the data; critical review of the literature; critical review of the manuscript.

Anna Karoline Fausto-Silva: Approval of the final version of the manuscript; elaboration and writing of the manuscript; obtaining, analyzing and interpreting the data; critical review of the literature; critical review of the manuscript.

Eliane Pedra Dias: Approval of the final version of the manuscript; conception and planning of the study; obtaining, analyzing and interpreting the data; critical review of the manuscript.

Sueli Carneiro: Approval of the final version of the manuscript; conception and planning of the study; elaboration and writing of the manuscript; obtaining, analyzing and interpreting the data; effective participation in research orientation; critical review of the literature; critical review of the manuscript.

## Conflicts of interest

None declared.
